# St. Jude Total Therapy studies from I to XVII for childhood acute lymphoblastic leukemia: a brief review

**DOI:** 10.1186/s43046-022-00126-3

**Published:** 2022-06-13

**Authors:** Abdallah A. Omar, Lina Basiouny, Ahmed S. Elnoby, Abeer Zaki, Mohamed Abouzid

**Affiliations:** 1 Department of Pharmaceutical Services and Sciences, Children’s Cancer Hospital Egypt (CCHE-57357), Cairo, Egypt; 2 Department of Clinical Research, Children’s Cancer Hospital Egypt (CCHE-57357), Cairo, Egypt; 3 Clinical Research Department, M.A.R.C for Medical Services and Scientific Research, Cairo, Egypt; 4 Clinical Research Department — DataClin, Cairo, Egypt; 5 Department of Physical Pharmacy and Pharmacokinetics, Poznan University of Medical Sciences, 6 Święcickiego Street, 60-781 Poznań, Poland

**Keywords:** Childhood acute lymphoblastic leukemia, ALL, St. Jude, Total Therapy

## Abstract

The therapy design of childhood acute lymphoblastic leukemia (ALL) has evolved over the past 60 years. The St. Jude Children’s Research Hospital has developed 17 treatment protocols from 1962 to 2017, aiming to have the most effective and least toxic treatment form. This review summarizes each protocol’s objectives, inclusion criteria, treatment phases, pharmacological agents, irradiation therapy, response criteria, risk stratification, type of relapse, and overall survival. The enhancement and successful application of preventive therapy for ALL and following a risk-stratified approach have progressively improved the cure rate of childhood ALL, with relatively few adverse sequelae. Moreover, St. Jude’s scientific theme serves as a reminder of the principal factor of research directed to a catastrophic disease such as ALL.

## Background

In 1962, the St. Jude Children’s Research Hospital (for simplicity hereafter referred to as St. Jude) opened its doors to treat one of the most common causes of mortality in childhood acute lymphoblastic leukemia (ALL). Previous clinicians tried to treat this fatal disease through multiple chemotherapeutic agents, yet still achieved transient responses and later relapsed [1]. The first controlled clinical trial in leukemia was carried out by Frei et al. and concluded in 1964 [2]. It aimed to demonstrate the impact of simultaneous administration of methotrexate (MTX) and mercaptopurine (6MP) on the remission period. Despite achieving more prolonged remission when administering MTX and 6MP than administering a single drug, all the patients eventually relapsed and died [2]. In addition, the team discovered a positive relationship between the symptomatic leukemic involvement of the central nervous system (CNS) and the length of remissions [2]. The symptoms included headache and vomiting due to high intracranial pressure, along with epileptiform seizures and cranial nerve palsies. It was possible only to temporarily control these complications by the intrathecal (IT) injection of MTX; however, due to frequent occurrence, external beam irradiation failed to eradicate them.

St. Jude’s first director, Dr. Donald Pinkel, introduced the “Total Therapy” series of studies for ALL, which successively revealed the possibility to cure the disease in a significant number of children. In St. Jude, the prime fundamental biological research and pioneering studies in childhood cancer served as vital components in designing an excellent scientific environment and integrative activities. Therefore, Pinkel started building Total Therapy studies based on the understanding that a single chemotherapeutic agent at a specific phase of the disease will not be convenient in another, and that maintaining treatment for 2–3 years with different drugs arranged in a specific subsequent manner can eradicate leukemic cells and maintain remission [1, 3–6]. During the past 60 years, the scientific importance and complexity of St. Jude Total Therapy have grown, and St. Jude has succeeded in reporting the results of sixteen Total Therapy studies. The hospital designed Total Therapy XVII in 2017 and remained in the recruitment phase.

This review summarizes findings and treatment protocols of the St. Jude Total Therapy studies (from I to XVII) for childhood ALL.

## Total Therapy studies I and II

In 1962, a pilot study was initiated to investigate whether the available chemotherapeutic agents could improve a 5-year cure rate. Total Therapies I and II had the main aim of rapidly achieving complete remission, evading resistance, complete eradication of residual leukemic cells from which resistant cells arose by introducing the idea of the combination of multiple effective therapeutic agents with a different mechanism of actions, full dose rather than half dose, and prevention of CNS leukemia through prophylactic irradiation therapy [7]. The era of Total Therapies I and II studies started on a limited number of patients (*N* = 15), not more than 18 years of age, diagnosed with acute lymphocytic or acute undifferentiated leukemia, not previously treated or treated with no more than 1 week of prednisone (PRD) and vincristine (VCR), and patients initially presented with CNS leukemia were also included [5, 6, 8].

There were three phases of the treatment; in phase I (i.e., remission induction), two drugs were used: PRD and VCR. Patients who did not exhibit a response were dropped from the study. Then, prophylactic craniospinal irradiation (CSI) with 500 rad of Cobalt-60 (^60^Co) was initiated as early as 30 days after complete remission to eradicate leukemic infiltrations. Immediately following CNS irradiation, phase II (i.e., intensive therapy) started with a combination of three drugs: 6MP, MTX, and cyclophosphamide (CYC). This intensive therapy phase aimed to reduce the leukemic cell population toward zero [9]. In phase III (i.e., the remission maintenance), the patients received VCR, higher intravenous (IV) doses of MTX, and lower doses of 6MP and CYC [10]. In the following year, 1963, the Total Therapy Study II was initiated with a few amendments added to the Total Therapy I regimen. In phase III, doses of VCR and CYC were decreased to half, and patients received either 6MP or MTX [1, 5, 6].

The evaluation of the response in St. Jude Total Therapy studies followed the criteria established by the Clinical Studies Panel of the Cancer Chemotherapy National Service Center [11]. Complete remission was defined as one wherein no evidence of disease could be found in the peripheral blood and the bone marrow, and all symptoms and signs of disease had subsided. In effect, a significant reduction of the total leukemic cell population existed in the untreated child and represented that time during the treatment when the size of the leukemic cell population was below detectable levels. Therefore, 13 out of 15 patients treated on Total Therapy studies I and II achieved complete marrow remission [12]. Two patients continued to have prolonged complete remission for 77 and 79 months and off-therapy duration of 15 and 36 months which was a glimpse of hope at the time [12].

In studies I and II, it was evident that 500 rad CSI irradiation had no noticeable effect on the relapse rate. The recurrence of leukemic cells in bone marrow, CNS, and viscera is known to relapse [13]. Therefore, myelosuppression was increased due to radiation, and the chemotherapy has demonstrated merely modestly effective [4, 6]. Therefore, many modifications were required to this protocol to increase overall survival (OS) rates and decrease toxicities [14].

## Total Therapy Study III

Study III aimed to increase the CSI irradiation from 500 to 1200 rad and then a combination chemotherapy regimen similar to Total Therapy I [1]. The study had 26 previously untreated patients below 16 years with ALL. By the end of phase I, marrow remission was achieved in 24 patients [15]. The use of combination therapy in children with leukemia carries the risk of immediate and long-range toxic reactions, as demonstrated in these studies. It is also possible that some children with 5-year cures will eventually experience relapse [15]. Also, 92% of the patients have complete remission, median hematological remission, and survival months were 22 and 35, respectively. The 5-year OS of patients was 6, while five patients were cured during the same period [15].

Moreover, 50% of the patients had initial relapses in the CNS [1], and this was the first time that initial relapses in the CNS were higher than those in the bone marrow. Hence, the radiation dose remained ineffective at controlling leukemia in the CNS [1]. This study established the main principle of therapy of simultaneous administration of multiple agents to get a leukemic effect at different cellular metabolic pathways for more therapeutic synergism and reduction of resistant strains to chemotherapeutic agents.

## Total Therapy Study IV

Study IV aimed to compare the treatment with chemotherapy at half-dose and full-dose levels due to the toxicity of the latter. Therefore, the chemotherapy program was comparable to the study III program except for dose modification in phase III. Moreover, CNS prophylaxis was not applied as in previous studies. However, if CNS leukemia developed as evidenced by ten or more leukemic cells per cubic millimeter (mm^3^) in the spinal fluid, this was treated with IT MTX, 12 mg/M^2^ once weekly or by radiation therapy, or both [1].

Fifty children aged between 11 months and 16 years with ALL were enrolled in the study from July 1965 to May 1967 [12]. Forty-five patients have completed remission, 3 patients failed to develop complete remission marrow, and two children died of infection [12]. Out of 45 patients, three children did not resume the study; one developed varicella, one received 4 months of PRD, and one denied the continuation of the treatment [12]. Therefore, after successful remission induction, 42 patients were divided (21; 21) randomly into either full-dosage (group A) or half-dosage (group B) chemotherapy groups with the same agents and schedule [1].

In group B, 20 patients developed CNS leukemia in a median of 5 to 7 months from complete remission marrow. Fifteen patients had CNS relapse — because of inadequate systemic chemotherapy — before hematologic relapse [12, 16]. Seven of the 50 children registered in this study died during its course. Three died during the induction phase: 2 of the infection and 1 of leukemia. Four died while on continuation chemotherapy; all four were in group A. Nonbacterial infection was the cause of death in 3 of the four children dying during remission. All 3 of these children had neutrophil levels greater than 2000/mm^3^ at the beginning of their terminal infections, which were reliable with broad experience [12].

The duration of initial complete remission in group A and group B was median = 35 and 6 months, respectively. Moreover, the median duration of initial hematologic remission in group A was 33 months compared with 16 months for group B.

Overall, 90% of the patients have complete remission (*N* = 45). The median duration of initial complete remission, hematological remission, and survival months were 15, 33, and 34 vs. 6, 16, and 24 for group A and group B, respectively. The aforesaid median duration was calculated in 50% remission and survival because 25% remission and survival values were insignificant. Four patients in group A continue in complete remission for 40 to 48 months and have been off treatment for 3 months to 1 year. Nine patients in group A and 4 in group B remain in continuous hematologic remission for 40 to 55 months [12]. The 5-year OS number of patients was 6, while five patients were cured within 5 years [15].

Concerning the relapse, 15 patients from group A have developed leukemia of the CNS (median = 10 months) following complete remission. Moreover, 10 of the 15 had CNS relapse (median = 10 months) before hematologic relapse. Therefore, CNS leukemia was the primary cause to terminate the complete remission status in this group. For group B, 20 developed CNS leukemia (median = 5–7 months) in a median time of 5 to 7 months from complete remission marrow. Fifteen of the 20 had CNS relapse before the hematologic relapse (median = 4 months). Study IV demonstrated that dosage reduction was associated with a pronounced reduction in infection during remission but with a shorter complete remission.

## Total Therapy Study V

An unpredicted optimistic result gradually emerged from initial studies I, II, and III. Maintaining long-term complete remissions was proved in two of the 15 children (studies I, II) and five of 26 children (study III). Hence, a chance was given for subsequent studies and driven to the construct study V [1]. Children with ALL were admitted to the study from December 1967 to July 1968 (*N* = 35; age median = 4.6 years, girls = 49%). Patients should not receive any therapy before except blood transfusions, cortisone for less than 7 days [14].

Due to higher incidence of CNS relapse, CNS therapy was restored with three major adjustments: (1) increasing irradiation dose from 1200 to 2400 rad, (2) irradiation of the cranium only since spinal irradiation may damage the marrow, and (3) administration of 5 doses of IT MTX [14]. Moreover, PRD and VCR were added every 10 weeks in phase III. The medications were administered at the maximum tolerated dose, which was possible when the total leukocyte count was higher than 3000/mm^3^ [14]. Only 50% of the dose was administered in case of leukocyte count was between 1000 and 2000/mm^3^. In addition, all the medications were stopped in cases of (1) leukopenia (total leukocyte count less than 1000/mm^3^), (2) fever lasting more than 2 days, (3) severe diarrhea or vomiting, and (4) mucosal ulceration [14].

Three children of 35 enrolled in the study died because of pseudomonas sepsis (*N* = 1; 10 days after attaining complete remission) and viral infections (*N* = 2; after 13 and 14 months of continuous complete remission). One patient developed systemic histoplasmosis after 26 months of continuous complete remission. Also, three patients were diagnosed with *Pneumocystis carinii* pneumonia (PCP), three developed herpes zoster, and two developed varicella, but all recovered without direct sequelae.

The results were remarkable among the 32 of 35 patients who attained remission. Ultimately, half of the patients went on to complete 3 years of therapy. All remained in remission after cessation of treatment became long-term survivors and were eventually declared cured. A subsequent randomized study confirmed the value of preventive CNS therapy [14]. There were two major relapses: hematological relapse (*N* = 5, after 12 to 27 months of continuous complete remission) and nervous system leukemia (*N* = 3, after 6 months of complete remission).

## Total Therapy Study VI

Study VI was conducted from 1968 to 1970 to define the importance of radiotherapy alone to prevent CNS leukemia. Also, it aimed to evaluate the effectiveness of a 1-week intensive chemotherapy course [17]. Children with ALL were admitted to the study (*N* = 110) if they did not receive any therapy before except blood transfusions, cortisone for 7 days or less, one injection of daunorubicin (DAN), and/or VCR similar to the same dose in study VI [17].

The study’s design was different from the previously mentioned ones. However, the three phases remain the same. The patients were divided mainly into two groups and four subgroups. In phase I, DAN was used for the first time with PRD and VCR. In phase II, patients were divided into two groups (first randomization: group A, received intensive chemotherapy — 7 days; group B, no intensive chemotherapy and enrolled directly to phase III). For group A, there was a 2-week delay between the last day of intensive chemotherapy and continuation therapy. After 4 weeks of complete remission, the second randomization started. Groups A/B were divided into four subgroups: A1/B1 with 2400 rads CSI — 4 weeks and A2/B2 without irradiation until demonstrable CNS leukemia [17]. In phase III, groups A and B received the same medication (3 years).

In this study, high-risk patients with ALL were those who had one or more of these findings in the initial recruitment stage, and they were disqualified: (1) leukocytosis was higher than 50,000/mm^3^ and (2) comprehensive leukemic infiltration of the viscera that negatively affects the response to therapy [18]. Overall, 86% of the enrolled children (group A = 45, group B = 49) developed complete remission marrow and completed both randomizations. The highest and lowest median duration of initial complete remission was 15 and 10 months for groups B1 and B2, respectively. However, the latter has the highest number of survivors (24; 92%), with a median survival of 19 months. These data show that 1-week course of intensive IV chemotherapy early in remission did not affect the duration of remission or survival; that lack of significant difference might be due to the combination chemotherapy used in both during the continuation phase equalizing the leukemic cell kill and masked the differences. CNS leukemia was prevented in 4.4% and 55% of children in groups A1/B1 and groups A2/B2, respectively. These results confirm the importance of 2400 rads CSI in preventing or delaying the onset of CNS leukemia. However, after developing CNS leukemia, the dose was insufficient to eliminate the disease. Moreover, hematologic relapse was evident in 5 patients in group A compared to 6 patients in group B.

## Total Therapy Study VII

The study aimed to improve event-free survival (EFS) by controlling meningeal leukemia using CNS irradiation-directed treatment and comparing two forms of CNS prophylactic therapy [3, 6, 13]. The study involved formerly untreated children (*N* = 110) below 20 years, leukocytes of 50,000/μL or more, and/or at least 10% French American-British (FAB) L2 blasts. Patients aged 2 to 10 years, bone marrow blasts of 10% with FAB L2 morphology, leukocytes less than 10,000/L [13, 19]. Complete remission was characterized by a lack of leukemia symptoms and physical findings with peripheral blood ≥ 500/mm^3^ neutrophils, platelets ≥ 75,000/mm^3^, and optimal hematopoiesis in cellular marrow and bone marrow containing less than 5% lymphoblasts.

Patients who attained complete remission were randomized into four groups (CM, CMVP, CS, CSVP) and immediately entered prophylactic CNS therapy. Two groups (CM, CMVP) were assigned to receive CSI and MTX: the first dose of IT MTX to be given on the 3rd day of radiotherapy and the subsequent doses given every 3–4 days for five doses and the last dose to be overlapped with the completion of radiotherapy [13, 19]. The remaining two groups (CS and CSVP) were exposed to CSI alone.

Following the CNS prophylactic therapy, continuation therapy has started. All groups were received continuation phase chemotherapy of 6MP, MTX, and CYC. Moreover, PRD and VCR were added only to CMVP and CSVP groups every 12 weeks after remission [13, 19].

Ninety-four patients achieved marrow remission; 5 had CNS leukemia (3 of 45 patients receiving IT MTX plus cranial irradiation and 2 of 49 receiving CSI). Thirty patients had a hematological relapse, and two patients died of viral myocarditis and disseminated varicella. Therefore, 57 of 94 patients were in continuous complete remission from 8 months to 28 months, while 77 survived for approximately 9–29 months (median = 15 months) [6]. Hematological relapse was the highest in study VII compared to V, VI, VIII (era 2), with approximately 50% of relapse to patients. The reason might be that a third agent was not obtained in the first phase or intensification phase [20]. Thirty out of 94 patients had a hematological relapse. Five out of 94 patients had CNS leukemia (3 of 45 patients receiving IT MTX plus cranial irradiation and 2 of 49 receiving CSI [6]. Testicular relapses occurred only in 4% of the patients in era II collectively (from V to IX). There is no evidence of testicular relapse in study XII [21]. The study showed that CSI alone and cranial irradiation with IT MTX effectively prevented CNS leukemia. However, CSI was associated with a higher incidence of leukopenia and chemotherapy interruption. Moreover, VCR-PRD pulses during the continuation phase did not affect the relapse rate or duration of remission.

## Total Therapy Study VIII

Study VIII was planned to examine the efficacy and toxicity of compilation therapy during remission and correlate aggressiveness of first 8 weeks of treatment and prolongation of remission duration in poor prognostic patients (this includes patients with CNS leukemia or mediastinal enlargement and patients who fail remission after 1 month of therapy) [6, 22]. Patients that had a mediastinal or other tumor mass were included if they had over 25% lymphoblasts in the bone marrow. The study consisted of 282 patients with a median age of 4 years (range: 3 months–19 years; 11% less than 2 and 22% above 10 years). There were 56% boys and 9% Black with median WBCs of 8.7 × 10^9^/L. Large mediastinal mass was found in 24 patients, and 9 had initial meningeal leukemia. Only 95 patients were screened for sheep erythrocyte receptors [22].

All patients immediately started the remission induction phase consisting of PRD, VCR, and asparaginase (ASP). Patients diagnosed with CNS leukemia (group A) received MTX, and those with anterior mediastinal mass (group B) were exposed to radiotherapy. Patients who did not achieve bone marrow remission after the remission induction period (group C) received daily PRD and DAN for three doses in weeks 5 and 6 and then for one dose in weeks 7 and 8. Patients who did not achieve complete remission after 8 weeks of chemotherapy were dropped from the study.

Those who achieved complete remission were immediately entered the CNS therapy. Patients presenting with CNS leukemia at diagnosis (group A) were exposed to a higher dose of CSI in 28 days along with MTX, while other patients were exposed to preventive CNS therapy consisting of cranial irradiation in 18 days and MTX. An additional phase of chemotherapy was given to all groups (A, B, C) without randomization after 1 week of CNS therapy completion.

Those groups were given MTX, 6MP, and CYC, while the remaining patients were randomly assigned to one of four regimens (Table 1). All chemotherapy agents were discontinued for randomized patients after 30 months of continuous complete remission and 36 months for patients given additional therapy. Patients who developed bone marrow relapse were dropped from the study. Patients who developed CNS relapse were treated with weekly IT MTX (12 mg/M^2^) for 4–6 weeks and then monthly. Moreover, testicular relapse was proved by biopsy and treated with irradiation (1500–2200 rads) [22].

**Table 1:** Treatment protocol St. Jude Total Therapy studies from I to XVI for childhood ALL

Protocol	Remission induction		Intensive chemotherapy	Prophylactic therapy	Continuation therapy
I 1962–1965	4–6 weeks^a^ ▪PRD (PO): 40 mg/M^2^/day — (divided into 3 to 4 doses) ▪VCR (IV): 1.5 mg/M^2^/week		1 week ▪6MP (IV): 1 gm/M^2^ — 3 days, followed by the following: ▪MTX (IV): 10 mg/M^2^/day — 3 days, followed by the following: ▪CYC (IV) 600 mg/M^2^ — once Meanwhile, PRD was discontinued gradually over 14 days	▪500 R ^60^Co CSI irradiation^n^	3 years ▪6MP (PO): 50 gm/M^2^/day ▪MTX (IV): 20 mg/M^2^/week ▪CYC (IV) 200 mg/M^2^/week ▪VCR (IV): 1 mg/M^2^/week (full or half dose)
II 1963–1966				▪500 R ^60^Co CSI irradiation^n^	3 years ▪6MP (PO): 50 gm/M^2^/day or MTX (IV): 20 mg/M^2^/week ▪CYC (IV): 200 mg/M^2^/2 weeks ▪VCR (IV): 1 mg/M^2^/2 weeks
III 1962–1965				11 days ▪1200 R ^60^Co CSI irradiation	3 years^b^ ▪6MP (PO): 50 gm/M^2^/day ▪MTX (IV): 20 mg/M^2^/day ▪CYC (IV): 200 mg/M^2^/week ▪VCR (IV): 1 mg/M^2^/week
IV 1965–1967				-	
V 1967–1968				2.5 weeks ▪2400 ^60^Co cranial irradiation ▪MTX (IT): 12 mg/M^2^ — twice weekly for five doses	3–5 years ▪6MP (PO): 50 gm/M^2^/day ▪MTX (IV): 20 mg/M^2^/day ▪CYC (IV): 200 mg/M^2^/week Every 10 weeks ▪PRD (PO): 40 mg/M^2^/day — 15 days ▪VCR (IV): 1.5 mg/M^2^/week — 3 doses
VI 1968–1970	4 to 6 weeks ▪PRD (PO): 40 mg/M^2^/day — (divided into 3 to 4 doses) ▪VCR (IV): 1.5 mg/M^2^/week ▪DAN (IV): 25 mg/M^2^/week		1 week^c^ Group A ▪6MP (IV): 1 gm/M^2^ — 3 days, followed by ▪MTX (IV): 10 mg/M^2^/day — 3 days, followed by ▪CYC (IV): 600 mg/M^2^ — once Group B ▪None	4 weeks^d^ Groups A1/B1 ▪2400 R ^60^Co CSI Groups A2/B2 ▪None	3 years ▪6MP (PO): 50 gm/M^2^/day ▪MTX (IV): 20 mg/M^2^/day ▪CYC (IV): 200 mg/M^2^/week Every 70 days ▪PRD (PO): 40 mg/M^2^/day — 15 days ▪VCR (IV): 1.5 mg/M^2^/week — 3 doses
VII 1970–1971	4 to 6 weeks ▪PRD (PO): 40 mg/M^2^/day — (divided into 3 to 4 doses) ▪VCR (IV): 1.5 mg/M^2^/week		-	Groups CM/CMVP ▪2400 R ^60^Co CSI ▪MTX (IT): 12 mg/M^2^ — twice weekly for five doses Groups CS/CSVP ▪2400 R ^60^Co cranial irradiation	2.5 years ▪6MP (PO): 50 gm/M^2^/day ▪MTX (IV): 20 mg/M^2^/week ▪CYC (IV): 200 mg/M^2^/week Groups CMVP/CSVP added (every 12 weeks) ▪PRD (PO): 40 mg/M^2^/day — 15 days ▪VCR (IV): 1.5 mg/M^2^/week — 3 doses
VIII^e^ 1972–1975	4 to 6 weeks ▪PRD (PO): 40 mg/M^2^/day — (divided into 3 to 4 doses) ▪VCR (IV): 1.5 mg/M^2^/week — 4 doses ▪ASP (IV): 10,000 units/M^2^/week — 2 doses (one on day 2 or 3 and the last one at day 8) ▪MTX (IV): 12 mg/M^2^/week^f^ ▪Site irradiation: 2500 — 3500 R^g^		-	Preventive: ▪2400 R ^60^Co cranial irradiation — 18 days ▪MTX (IT): 12 mg/M^2^ — twice weekly for five doses Therapeutic Group A above ▪3000 R ^60^Co CSI — 28 days ▪MTX (IT): 12 mg/M^2^ — twice weekly for four doses	2.5 to 3 years Groups A/B/C ▪6MP (PO): 50 gm/M^2^/day ▪MTX (IV): 20 mg/M^2^/week ▪CYC (IV): 200 mg/M^2^/week The rest of the patients were randomized as follows: Group1: MTX Group2: MTX + 6MP Group3: MTX + 6MP + CYC Group4: MTX + 6MP + CYC + CYT^θ^
IX 1975–1979	4 weeks ▪PRD (PO): 40 mg/M^2^/day ▪VCR (IV): 1.5 mg/M^2^/week — 4 doses ▪DAN (IV): 25 mg/M^2^/week Patients are randomized to receive either the following: ▪ASP (IV): 10,000 lU/M^2^ — (2 doses, on days 3 and 9) OR ▪ASP (IV): 10,000 lU/M^2^ — twice/week for 4 doses ▪CYT (IV): 300 mg/M^2^ — twice/week for 4 doses		15–30 months^h^ Group PVD ▪PRD (PO): 40 mg/M^2^/day — 2 weeks ▪VCR (IV): 1.5 mg/M^2^/week — 3 weeks ▪DAN (IV): 25 mg/M^2^/week — 2 weeks Group VC ▪VCR (IV): 1.5 mg/M^2^/week — 3 weeks ▪CYC (IV): 300 mg/M^2^/week — 3 weeks Group VM-26 + ara-C ▪VM26 (IV): 165 mg/M^2^ twice weekly for 2 doses ▪CYT (IV): 300 mg/M^2^ twice weekly for 2 doses Group ASP ▪ASP (IM): 10,000 IU/M^2^ daily for 5 doses	18 days^i^ ▪2400 R ^60^Co cranial irradiation (age adjusted) ▪MTX (IT): 12 mg/M^2^, 5 times (maximum dose, 15 mg)	2.5 years ▪MTX (IV): 20 mg/M^2^/week ▪6MP (PO): 50 mg/M^2^/day
X 1979–1983	4 weeks ▪PRD (PO): 40 mg/M^2^/day ▪VCR (IV): 1.5 mg/M^2^/week ▪ASP (IV): 10,000 units/M^2^ (on days 4, 8, 12, 15) ▪MTX (IT): 12 mg/M^2^ (max. 12 mg, on days 15 & 29) Followed by 78 weeks of continuation therapy: ▪6MP — once a day and MTX — once a week with 5 intermittent doses of VM26 and CYT every 10 weeks only until week 52 ▪Preventive CNS therapy after 1 year of persistent remission		High-risk relapse — add the following: ▪VM26 (IV): 150 mg/M^2^ ▪CYT (IV): 300 mg/M^2^ — twice weekly pre- and post-conventional induction therapy	▪1800 R ^60^Co cranial irradiation ▪MTX (IT): 5 doses 12mg/M^2^	Treatment A — 3 weeks ▪MTX (IV): 200 mg/M^2^ followed by 24-h infusion 800 mg/M^2^ ▪MTX (IT): 12 mg/M^2^ ▪Leucovorin rescue (IV)^j^: 30 mg/M^2^, 12 and 18 h post-MTX infusion and 3 mg/M^2^ orally/12 h for 3 doses with IV hydration (5% dextrose and 0.2% NaCl, 100 mL/M^2^/h) and urinary alkalinization (NaHCO_3_, 1 g/M^2^ orally/6 h) given 2 h before each infusion of HDMTX Then 120 weeks of the following: ▪6MP (PO): 50 mg/M^2^ — daily ▪MTX (PO): 25 mg/M^2^ weekly with intermittent shots of high dose ▪MTX every 6 weeks until week 72 OR Treatment B — 72 to 120 weeks 78 weeks of continuation therapy ▪6MP — once a day and MTX — once a week with 5 intermittent doses of VM26 and CYT every 10 weeks only until week 52 and preventive CNS therapy after 1 year of persistent remission RTSC group ▪MTX (IT): 12 mg/M^2^ (every 3 months) HDMTX group ▪HDMTX + IT-MTX ▪6MP + IT-MTX ▪Doxo + Cyclo ▪VM26 + CYT ▪MTX (IV): 200 mg/M^2^ — followed by 24-h IV every 1.5 months for 18 months ▪MTX (IT): 12 mg/M^2^ — 12 weeks for 30 months ▪6MP (PO): 50 mg/M^2^ ▪CYC (PO): 100 mg/M^2^/day ▪DOX (IV): 30 mg/M^2^ ▪VM26 (IV): 150 mg/M^2^ ▪CYT (IV): 300 mg/M^2^ — every 2 week
XI 1984–1988	4–6 weeks ▪PRD (PO): 40 mg/M^2^/day — (divided into 3 to 4 doses) ▪VCR (IV): 1.5 mg/M^2^/week ▪DAN (IV): 26mg/M^2^ ▪ASP (IV): 1000000 units/M^2^ ▪VM26 (IV): 200 mg/M^2^ ▪CYT (IV): 300 mg/M^2^		▪HDMTX (IV): 2g/M^2^ — 2 doses/week	High risk ▪13–15 doses of TIT ▪1800 R ^60^Co cranial irradiation ▪2400 R initially CNS infiltration Low risk ▪9 doses of TIT only	High riskn Four pairs of drugs rotated every week or every 6 weeks ▪VP16 and CYC ▪6MP and MTX ▪VM26 and CYT ▪PRD and VCR Low risk^n^ ▪Randomized to take the four pairs of drugs OR ▪6MP and MTX — 3 weeks ▪PRD and VCR — 1 week
XII 1988–1991				3 weeks High risk ▪18–20 doses TIT ▪1800 R ^60^Co CSI ▪2400 R initially CNS infiltration Low risk ▪13 doses of TIT	Patient stratified and randomized to talk individualized dose or conventional dose ▪HDMTX ▪6MP (given as permanent drugs 120 weeks) ▪MTX (given as permanent drugs 120 weeks) ORξ ▪VM26 ▪CYT ▪6MP ▪MTX^o^
XIIIA 1991–1994	Pre-induction chemotherapy (2–4 days) ▪HDMTX (IV): 1 mg/M^2^ OR ▪LDMTX: 30 mg/M^2^ every 6 h before the induction	Induction chemotherapy ▪The same of XI except; VP16 was given instead of VM26	2 weeks ▪HDMTX (IV): 2g/M^2^ — 2 doses/week ▪6MP (PO): 75 mg/M^2^/day	3 weeks High risk ▪22 to 26 doses TIT ▪1800 R ^60^Co cranial irradiation ▪2400 R initially CNS infiltration Low risk ▪15 doses of TIT only	120 weeks High risk^n^ ▪CYC + VP16 ▪MTX + 6MP ▪MTX + CYT ▪PRD + VCR + ASP ▪VP16 + CYC ▪HDMTX + 6MP ▪CYT + VP16 ▪PRD + VCR + ASP Low risk^n^ ▪6MP + MTX (120 weeks) ▪HDMTX pulses/8 weeks ▪PRD +VCR/4 weeks Reinduction weeks (32–37) ▪PRD (PO): 40 mg/M^2^/day — (divided into 3 to 4 doses) ▪VCR (IV): 1.5 mg/M^2^/week ▪DAN (IV): 26mg/M^2^ ▪ASP (IV): 1000,000 units/M^2^ ▪VM26 (IV): 200 mg/M^2^ ▪CYT (IV): 300 mg/M^2^ ▪HDMTX (IV): 2g/M^2^ — 2 doses/week
XIIIB 1994–1998	Pre-induction chemotherapy (2–4 days) ▪6MP (1 g/M^2^) OR ▪HDMTX (IV): 1 g/M^2^ ▪6MP: 1 g/M^2^ OR ▪LDMTX (PO): 30 mg/M^2^ ▪6MP 1 g/M^2^			3 weeks High risk ▪26 doses TIT ▪1800 R ^60^Co cranial irradiation ▪2400 R initially CNS infiltration Low risk ▪13 doses of TIT only	The same of XIIIA except the following: ▪PRD was replaced with DEX ▪ASP was given only during the reinduction phase
XIV 1998–1999			2 weeks High risk ▪HDMTX (IV): 5 g/M^2^ — 2 doses /week ▪6MP (PO): 75 mg/M^2^/day Low risk ▪HDMTX (IV): 2.5 g/M^2^ — 2 doses/week ▪6MP (PO): 75 mg/M^2^/day	During the entire treatment period High risk ▪23 doses of TIT Low risk ▪16 doses of TIT	The same of XIIIB except^p^ the following: Low risk ▪HDMTX (PO): 2.5 g/M^2^ High risk ▪HDMTX (PO): 5 g/M^2^ Reinduction phase ▪At day 1, 8 PEG-ASP + idarubicin ▪At day 15, PEG-ASP only ▪VCR (IV): 1.5 mg/M^2^/week ▪DEX (PO): 8 mg/M^2^/day
XV^k^ 2000–2007	4–6 weeks ▪PRD (PO): 40 mg/M^2^/day ▪VCR (IV): 1.5 mg/M^2^/week — divided into 4 doses ▪DAN (IV): 25 mg/M^2^/week ▪L-ASP (IM): 10,000 units/M^2^ divided into 5 doses ▪CYC (IV): 1000 mg/M^2^ — once ▪CYT (IV): 75 mg/M^2^ — 8 doses ▪6MP (PO): 60 mg/M^2r^ ▪Imatinib (PO): 40 mg/M^2l^		Consolidation phase (8 weeks) ▪HDMTX targeted dose depending on risk status, days 1, 15, 29, and 43 ▪6MP (PO): 50 mg/M^2^/day, days 1–56 Intensive chemotherapy^m^ ▪DEX (PO): 20 mg/M^2^ ▪CYT (IV): 2 g/M^2^ ▪VP16 (IV): 100 mg/M^2^ ▪L-ASP (IM): 25,000 units/M^2^ ▪1 dose of IT chemotherapy	Intrathecal chemotherapy, dose age dependent^ρt^ Low risk with CNS1 ▪13 doses of TIT Low risk with CNS2 ▪18 doses of TIT Standard risk cases with CNS1 ▪16 doses of TIT Standard risk cases with CNS2 ▪18 doses of TIT Standard/high-risk cases or CNS3 ▪24 doses of TIT	120 weeks High risk ▪ASP (IM): 25,000 units/M^2^/day ▪6MP (PO): 50 mg/M^2^ divided into 7 doses ▪DEX (PO): 12 mg/M^2^/day ▪VCR (IV): 2 mg/M^2^ ▪DOX (IV): 30 mg/M^2^ ▪3 cycles reinduction I and 3 cycles reinduction II Low risk ▪6MP (PO): 75 mg/M2 plus ▪MTX (IV or IM): 40 mg/M^2^ ▪DEX (PO): 8 mg/M^2^ ▪VCR (IV): 2 mg/M^2^ alternating with the following: ▪3 cycles reinduction I and 3 cycles reinduction II
				Reinduction after continuation therapy	
				Reinductions I and II for low risk ▪DEX (PO): 8 mg/M^2^/day ▪VCR (IV): 1.5 mg/M^2^/week (max. 2 mg) ▪L-ASP (IM): 10,000 units/M^2^ — once a week ▪DOX (IV): 30 mg/M^2^/week	Reinduction I for standard/high risk ▪DEX (PO): 8 mg/M^2^/day ▪VCR (IV): 1.5 mg/M^2^/week ▪DOX (IV): 30 mg/M^2^ ▪L-ASP (IM): 25,000 units/M^2^ — divided into 3 doses Reinduction II for standard/high risk ▪As mentioned in reinduction I plus high-dose CYT (IV): 2 gm/M^2^
XVI^k^ 2007–2017	4–6 weeks ▪PRD (PO): 40 mg/M^2^/day^q^ ▪DEX (PO): 10 mg/M^2^/day^q^ ▪VCR (IV): 1.5 mg/M^2^/week — divided into 4 doses ▪DAN (IV): 25 mg/M^2^/week ▪L-ASP (IM): 10,000 units/M^2^ divided into 5 doses ▪CYC (IV): 1000 mg/M^2^ — once ▪CYT (IV): 75 mg/M^2^ — 8 doses ▪6MP (PO): 60 mg/M^2^ ▪Imatinib (PO): 40 mg/M^2l^			Intrathecal chemotherapy, dose age dependent^st^ Low risk with CNS1: ▪13 doses of TIT low risk with CNS2 ▪17 doses of TIT Standard risk cases with CNS1 ▪16 doses of TIT Standard-risk cases with CNS2 ▪20 doses of TIT Standard-/high-risk cases or CNS3 ▪27 doses of TIT	120 weeks Low-risk patients ▪Two reinduction cycles ▪DEX (PO): 8 mg/M^2^ ▪VCR (IV): 2 mg/M^2^ Standard or high risk ▪PEG-ASP (IV): 2,500 vs. 3,500 units/M^2^ — 15 doses ▪Intermittent doses of DOX (IV): 30 mg/M^2^ ▪VCR (IV): 2 mg/M^2^ ▪DEX (PO): 12 mg/M^2^ ▪Two reinduction cycles ▪6MP (PO): 50 mg/M^2^ ▪MTX (IV): 40 mg/M^2^
				Reinduction after continuation therapy	
				Reinduction I for low risk ▪DEX (PO): 8 mg/M^2^/day (t.i.d.) ▪VCR (IV): 1.5 mg/M^2^/week — divided into 3 doses ▪PEG-ASP (IV): 2,500 or 3,500 units/M^2^ -divided into 2 doses ▪DOX (IV): 30 mg/M^2^/day ▪IT chemotherapy, dose age dependent, day 1 Reinduction II for low risk ▪DEX (PO): 8 mg/M^2^/day ▪VCR (IV): 1.5 mg/M^2^/week — divided into 3 doses ▪PEG-ASP (IV): 2,500 or 3,500 units/M^2^ — divided into 2 doses ▪IT chemotherapy, dose age dependent, day 1 From week 21 to the end of the therapy ▪6MP (PO): 75 mg/M^2^ ▪MTX (IV or IM): 40 mg/M^2^ ▪6MP (PO): 75mg/M^2^ — intermittent ▪DEX (PO): 8 mg/M^2^ ▪VCR (IV): 2 mg/M^2^ (max. 2 mg)	Reinduction I for standard/high risk ▪DEX (PO): 8 mg/M^2^/day — t.i.d. ▪VCR (IV): 1.5 mg/M^2^/week — divided into 3 doses ▪DOX (IV): 30 mg/M^2^ ▪PEG-ASP (IV): 2,500 or 3,500 units/M^2^ Reinduction II for standard/high risk and MLL infants ▪DEX (PO): 8 mg/M^2^/day — t.i.d. ▪VCR (IV): 1.5 mg/M^2^/week ▪PEG-ASP (IV): 2,500 or 3,500 units/M^2^ ▪High-dose CYT (IV): 2 gm/M^2^ ▪IT chemotherapy, dose age dependent^s^ From week 21 to the end of the therapy ▪PEG-ASP (IV): 2,500 vs 3,500 units/M^2^ every other week separated by the following: ▪CYC (IV): 300 mg/M^2^ plus ▪CYT (IV): 300 mg/M^2^ ▪6MP (PO): 75mg/M^2^ ▪MTX (IV or IM): 40 mg/M^2^ ▪DEX (PO): 12mg/M^2^ ▪VCR (IV): 2 mg/M^2^ (max. 2 mg) ▪PEG-ASP (IV): 2,500 and 3,500 units/M^2^

^a ^study III had 42 days, ^b^ group IVa took the full dose while group IVb took the half dose, ^c ^1st randomization, only group VIa administered the intensive chemotherapy, ^d ^2nd randomization, only groups VIa1 and VIb1, ^e ^if marrow not in remission after 1 month, PRD (PO): 40 mg/M^2^/day + DAN (IV): 25mg/M^2^/day-3 doses, ^f ^if the patient had CNS leukemia, group a, ^g ^If the patient had mediastinal mass, group b; θ–50 mg/M^2^/week, ^h ^late intensification after 15–30 months from complete remission. Patients are assigned into 4 groups, ^i ^initiated either after complete remission is achieved or after the intensive phase, ^j ^from the protocol development perspective, there is a lack of information if leucovorin rescue was reported in each following protocol, however, from the toxicity perspective, HDMTX must be followed by leucovorin administration, ^k ^intrathecal therapy will be administered on days 1 and 19, dose age dependent. Patients with a high risk of CNS relapse will receive additional IT treatments on days 8 and 26, ^l ^twice daily for Ph-positive patients starting from day 22, ^m ^for high risk only, before bone marrow transplantation, ^n ^as originally reported, ^o ^given interchangeably every 6 weeks for 5 courses of each (first year), ^p ^the period of the continuation phase was 120 weeks for girls and 146 weeks for boys, ^q ^dexamethasone will replace prednisone in patients with early T-cell precursor immunophenotype, ^r^replaced by thiopurine in thiopurine S-methyltransferase deficiency or hematotoxicity, ^s^ day 1: MTX + hydrocortisone + CYT doses and routes are age-dependent MTX 6, 8, 10, or 12 mg; hydrocortisone 12, 16, 20, or 24 mg; and CYT 18, 24, 30, or 36 mg for age 1, 1–1.99, 2–2.99, and 3 years, respectively, ^t^ CNS irradiation is removed except in refractory CNS leukemia or patients with lymphoblasts in the CSF. ALL, acute lymphoblastic leukemia, *ASP *asparaginase, *CNS *central nervous system, *CSF *cerebrospinal fluid, *CS *craniospinal irradiation, *CYC *cyclophosphamide, *CYT *cytarabine, *DAN *daunorubicin, *DEX *dexamethasone, *DOX* doxorubicin, *HDMX *high-dose methotrexate, *IV* intravenous, *L-ASP-L* asparaginase, *LLy* lineage lymphoblastic lymphoma, *MTX* methotrexate, *PCP*, Pneumocystis carinii pneumonia, *PEG* polyethylene glycol, *PO *oral, *PRD *prednisone, *RBCs *red blood cells, *RTSC *cranial irradiation/sequential chemotherapy, *t.i.d.* three times a day, *TIT *triple intrathecal therapy, *VCR* vincristine, *VM26 *teniposide, *VP16* etoposide, *WBCs* white blood cells

Out of 282 subjects who entered this study, 268 reached complete remission. Thirteen patients failed before completing 1 month of therapy (six of them died of sepsis, one patient reached remission but died of pneumonia and was considered an induction failure). Initial complete remission was achieved in 52% of patients, and 65% survived. PCP, meningococcemia, and leukoencephalopathy ended initial complete remission in 10 patients [22]. Several relapse incidences were reported: 21 patients – CNS; 66 patients – bone marrow; and 5 patients – testicular [22, 23].

## Total Therapy Study IX

Total Therapy Study IX was designed to assess two major therapy amendments: (a) adding a fourth drug to a standard induction regimen and (b) incorporation of a consolidation phase (then considered intensive) immediately after 3-drug remission induction to prevent resistant clones [11, 23, 24]. From December 1975 to May 1979, previously untreated patients are less than 20 years old with differentiated ALL. The study included 290 patients, 53% boys, and 8% Black patients. The median age in the study was 5 years (range = 2 months–19 years), 51 patients (18%) were 2 years old or less, and 73 (26%) were more than 10 years. The leukocyte median was 13 × 10^9^/L (range = 1–880 × 10^9^/L). Twenty-four patients had a mediastinal mass, and 12 had CNS leukemia initially. Sheep erythrocyte-rosette formation was positive in the bone marrow blasts of 13% of patients [21, 24]. Also, patients were stratified according to their presenting features, for instance, high-risk features (leukocytic count > 100 × 10^3^/mm^3^, CNS/mediastinal mass involvement, positive erythrocyte rosette test) and standard risk features (leukocytic count < 100 × 10^3^/mm^3^, no CNS/mediastinal mass involvement, negative erythrocyte rosette test) [25, 26].

In addition to PRD, VCR, and DAN, patients were randomized to receive either ASP only during the induction or intensive therapy of ASP and cytarabine (CYT) right after remission induction. In phase II, the patients were assigned into four groups (Table 1), and in the continuation therapy, only MTX and 6MP were administered.

Complete remission was attained in 92% of patients. Overall, 102 patients remained in initial complete remission, 117 were in initial hematological remission, and 149 survived [24, 26]. Sixty-five patients remained in continuous complete remission for 14 to 30 months, and 75% of them remained in continuous complete remission for 4.6 to 6.3 years [24].

Out of 277 patients, extramedullary relapses ended initial complete remissions in 37 children. Twenty-three relapses occurred in the CNS, fourteen patients had testicular relapses, and seven had subclinical testicular leukemia. All four patients with the manifested testicular disease had successive hematological or CNS relapses during the continuation phase. Sixteen patients had hematological and CNS relapses, and 91 patients had hematological relapse [24]. Seven patients had occult disease, and five of them have been off treatment for 9 to 43 months. One patient died of Epstein-Barr viral infection in remission, and one developed a hematological relapse 9 months later. Three children had testicular leukemia after therapy for 16, 28, and 41 months, respectively [24].

In the early effective therapy, 5-year OS tremendously increased from 9 to 36% due to CNS preventive approach, which significantly boosted patients’ survival in this era [6]. Five-year EFS for this era (studies from V to IX) collectively was 36% (standard error (SE) = 2%) [6].

## Total Therapy Study X

Due to limited intensification, Total Therapy Study IX has failed to improve the remission rate. Therefore, Total Therapy Study X aimed to test if high-dose MTX (HDMTX, 1 g/M^2^) will effectively sustain complete remission in ALL patients along with prophylactic sequential chemoradiotherapy. Besides, study X also tested the combination of teniposide (VM26) and CYT in continuation therapy to eliminate resistant leukemic cells [6, 26, 27].

Patients enrolled in this study (*N* = 330) had to be newly diagnosed with ALL or undifferentiated leukemia, less than 18 years old, not previously treated (patients who were treated for more than 1 week or were excluded). Also, patients were not enrolled if they had other treatment than glucocorticoids, VCR, or mediastinal irradiation [26, 28]. Patients were stratified according to the study IX scheme [25, 26].

Patients received the 4-week standard induction therapy of PRD, VCR, ASP, and MTX [25, 26]. Those with a high risk of relapse added VM26 and CYT to their therapy. All patients received modified doses to sustain leukocytic counts to approximately 2–4 × 10^9^/L. The patients were randomized into two groups starting from phase II: HDMTX and RTSC (Table 1) [6, 21, 23].

Overall, 49 patients had bone marrow relapse, six had CNS relapse, and eight testicular relapses. Also, secondary myeloid leukemia appeared in four patients, three patients relapsed with extramedullary masses, and one patient relapsed with anaplastic astrocytoma [23, 26].

HDMTX group has significantly (*P* < 0.001) better survival results than the RTSC group, and over 90% of HDMTX patients have survived for at least 6 years. The 5-year EFS of study X was better than era 2 (studies from V to IX), with values of 53 (SE = ±2) and 36 (SE = ±2), respectively [6, 26].

## Total Therapy Study XI

Total Therapy XI assessed early intensified induction therapy proving the Goldie-Coldman model of tumor cell kinetics and drug resistance which is a mathematical model that predicts tumor cell sensitivity and the maximum chance for cure when all available drugs are given simultaneously, early in therapy, and in the highest tolerable dose [27, 29]. Patients enrolled in this study (*N* = 358) had similar inclusion criteria as in study X [28, 29] which include high-risk features (leukocytic count initially > 100 × 10^3^/mm^3^) or involvement of 2 or more of the following unfavorable features (age < 2 years old or ≥ 10 years old, presence of chromosomal translocations, DNA index < 1.16, non-white race, leukocytic count > 25 × 10^9^/L, CNS involvement/extramedullary leukemia at presentation, CALLA −ve T or B immunophenotype, morphological examination of BM ≥ 5%) [29, 30].

To overcome the limited intensification of therapy in the past era, Total Therapy Study XI used early intensified chemotherapy with six chemotherapeutic agents during induction (PRD, VCR, ASP, DAN, CYT, VM26) followed by consolidation of 2-week HDMTX [30]. After intensification therapy, patients were randomized according to their risk stratification:

i. Lower-risk patients were randomized to receive either antimetabolite-based treatment (6MP and MTX) or alternating four pairs of chemotherapeutic agents (VP16 and CYC; VM26 and CYT; 6MP and MTX; PRD and VCR) alternated weekly for 120 weeks according to the Goldie-Coldman hypothesis.
ii. High-risk patients were randomized to receive the same four pairs of alternating chemotherapeutic pairs either weekly for 120 weeks or every 6 weeks for 120 weeks and then treatment with triple intrathecal therapy (TIT) consisting of MTX 12 mg, CYT 36 mg, and hydrocortisone 24 mg for all patients instead of MTX only (on days 2, 22, 43 of remission induction and additionally on days 8 and 15 for initial CNS leukemia) for every 8 weeks of first-year continuation therapy. Cranial irradiation is indicated (at year 1) for high-risk patients with a lower dose of 1800 rad, while the presence of CNS leukemia initially necessitated the use of higher doses (2400 rad). Cranial irradiation was given at 1 year for 63% of patients either initially diagnosed CNS infiltration or high risk [3, 21, 28, 31].

CNS and hematologic relapses were the most common among patients treated with Total Therapy Study XI. There were 17 induction failures, and 341 patients were in complete remission. Fifty-two cases of isolated or combined hematological relapse have been noted. There was only one case of testicular relapse, 20 CNS relapses, and 11 secondary malignancies. The secondary acute myeloid leukemia (AML) was higher in high-risk patients treated with four pairs of drugs rotated every 6 weeks than others and three extramedullary relapses. The cumulative risk at 10 years for isolated CNS relapse was 5.9 ± 1.3% and for CNS relapse was 7.3 ± 1.4%.

Early intensification of therapy and administration of rotational non-cross-resistant chemotherapeutic pair of drugs in the era of Total Therapy Study XI tremendously improved the estimated 5-year EFS from 53 to 71%, which was an outstanding achievement in the treatment of ALL [6].

## Total Therapy Study XII

In the Total Therapy Study XII era, pharmacokinetics played a vital role in individualizing doses in therapy to reduce systemic toxicities, thus enhancing treatment outcomes for ALL patients, which was the main objective of the Total Therapy Study XII. Patients enrolled in this study (*N* = 330) must be newly diagnosed with ALL or undifferentiated leukemia, less than 18 years old, not previously treated (patients who were treated for more than 1 week were excluded). Patients were not enrolled if they had other treatment than glucocorticoids, VCR, or mediastinal irradiation [28, 29]. They were stratified as high risk in case of chromosomal translocations as mixed-lineage leukemia (MLL) gene rearrangement or t (9; 22), and other than that, they are considered intermediate risk despite age or leukocytic count [32].

The study had two phases: induction and consolidation, similar to study XI. The patients who were in complete remission were stratified and then randomized to receive either individualized that is based on the area under plasma concentration-versus-time curve dose or conventional that depends on body surface area dose of continuation phase drugs (HDMTX, 6MP, and MTX) or (VM26, CYT, 6MP, and MTX) were given interchangeably every day for 6 weeks for five courses of each at the first year of continuation phase. 6MP and MTX were given as permanent drugs for 120 weeks of continuation. The CNS-directed therapy was 13 and 18–20 doses of TIT for low- and high-risk patients.

Additionally, high-risk patients had CSI (1800 rad); however, the CSI (2400 rad) for patients initially diagnosed with CNS leukemia was given from week 59–61 [28, 29, 32]. The Philadelphia (Ph)-positive patients who achieved complete remission in the studies (XI, XII) were eligible for allogeneic bone marrow transplantation [30]. Three out of six patients were eligible for hematopoietic stem cell transplantation due to Ph chromosome-positive leukemia [3, 28–30].

There were six induction failures and 182 patients in complete remission, 30 isolated or combined hematological relapses, two isolated testicular relapses, 11 secondary malignancies, and one extramedullary relapse. However, the literature shows that the low intensity of chemotherapy and the testicular irradiation were not adequate for patients initially diagnosed with testicular infiltration [27–29]. The cumulative risk at 10 years for isolated CNS relapse was 10.4 ± 2.3% and for CNS relapse was 14.9 ± 2.7%.

Total Therapy XII has the highest record of CNS relapses. Therefore, 10-year EFS decreased from 69.6 ± 2.4% during Total Therapy XI to 61.2 ± 3.6% during Total Therapy XII, while 10-year OS increased from 76.5 ± 2.2% in Total Therapy XI to 78.7 ± 3% in Total Therapy XII. In conclusion, there was no significant difference in treatment outcome between Total Therapies XI and XII [28].

## Total Therapy studies XIIIA and XIIIB

The study XIIIA aimed to prevent early CNS relapse and improve the outcome and effectiveness of overall ALL therapy. The study XIIIB was the first trial to apply the pharmacogenetics effect on the protocol’s drugs and the conformation of TIT efficacy and elimination of cranial irradiation for the following studies [3, 28, 29]. The patient must be diagnosed as ALL and should not receive more than 1 week of treatment other than VCR, glucocorticoids, and mediastinal irradiation [28, 29].

The criteria for low-risk cases was the leukocyte count below 50 × 10^9^/L, 1 to 9 years, DNA index from 1.16 to 1.60, CNS1 status (WBCs < 5/μL without cerebrospinal fluid (CSF) leukemic blasts) [33, 34], and good early treatment response) morphological examination of bone marrow < 5%) at day 15 of induction phase in the absence of T-cell leukemia, testicular infiltration and t(1:19), t(4;11), and t(9:22) of the Ph chromosome and MLL gene rearrangement, and the other patients stratified as high risk [29, 34–37].

The study XIIIA consisted of four phases: induction, consolidation, continuation, and reinduction. The induction phase was the same as studies XI and XII, the criteria for the patients were stratified to take HDMTX or low-dose MTX (LDMTX) every 6 h was given before the induction phase, and VP16 was given instead of VM26. After achieving complete remission, the consolidation phase was started, and patients received HDMTX and 6MP for 2 weeks. The continuation therapy was based on risk stratification and was risk-directed therapy. Every drug group was rotated every week, respectively, but the ASP was discontinued after 28 weeks, and high-dose MTX was discontinued after 1 year (Table 1). The reinduction phase from week 32 to 37 included the same drugs in primary induction therapy and HDMTX. The CNS therapy for low-risk patients was 15 doses of TIT and for high-risk patients was 22 to 26 doses of TIT plus cranial irradiation (from week 56 to 59) with doses of 1800 rad and 2400 rad for patients initially diagnosed as CNS leukemia. Only 22% of patients with high risk or initially CNS3 ( WBCs > 5/μL without CSF leukemic blasts) took the TIT and cranial irradiation during continuation [28–30, 33, 35, 38, 39].

The study XIIIB was similar to XIIIA with minor modification; the patients were stratified according to the risk criteria and randomized to upfront therapy with 6MP alone, HDMTX + 6MP, or LDMTX+ 6MP followed by 6 weeks of induction phase as previous just achieving complete remission consolidation phase was started. Patients received HDMTX (2 g/M^2^) 2 doses per week and daily 6MP (75 mg/M^2^) for 2 weeks. The continuation therapy was the same as study XIIIA, except the PRD was replaced with dexamethasone (DEX) during the continuation phase for all patients, and the ASP was given only during the reinduction phase. The CNS-directed therapy was the same as 13 A except for only 12% of the patient with T-cell ALL and WBCs > 100 × 10^9^/L or initially diagnosed as CNS3, the total number for TIT doses from 13 for low risk to 26 for high risk. The allogenic bone marrow transplantation was done for some eligible cases of high-risk patients [28–30, 35, 36, 38, 40].

The outcome of study XIIIA (*N* = 165 patients) had an OS estimate of 83.0 ± 2.9% at 5 years, 78.2 ± 3.2 at 10 years, 76.9 ± 5.2% at 15 years, and an EFS 76.9 ± 3.3% at 5 years and 71.5 ± 3.5% at 10 years 70.2 ± 5.8% at 15 years. Also, the outcome of study XIIIB (*N* = 247 patients) had an OS estimate 83.7 ± 2.5% at 10 years and an EFS 80.8% ± 2.6% at 5 years and 77.6 ± 2.9% at 10 years [28, 29, 37].

In St. Jude study XIIIA, there were three induction failures of 165 patients, 162 patients were in complete remission, 23 isolated or combined hematological relapse, two isolated testicular relapses, one combined testicular relapse, two isolated CNS relapses, 12 secondary malignancies, and one extramedullary relapse. The rate of secondary myeloid malignancies was higher than in the previous Total Therapy studies; the cumulative risk at 15 years for isolated CNS relapse was 1.2 ± 0.9% and for CNS relapse was 4.9 ± 1.7 %. For study XIIIB, five induction failures of 247 patients, 242 patients were in complete remission, 19 hematological relapse, one isolated testicular relapse, four isolated CNS relapse, four combined CNS relapse, and eight secondary malignancies; the cumulative risk at 10 years for isolated CNS relapse was 1.7 ± 0.8% and for total CNS relapse was 3.0% ± 1.1% [28, 29, 36, 37].

One of the most important prognostic factors is early treatment response. Its evaluation depends on morphological examination of bone marrow, or the level of minimal residual disease (MRD) by flow cytometry must be less than 0.01%, which has been a significant prognostic factor [28, 29, 36].

## Total Therapy Study XIV

The study was conducted to determine if irradiation therapy for CNS could be safely omitted and replacement with early intensification chemotherapy and TIT and determine the optimal dose of MTX in a different type of ALL [41, 42]. The patient must be diagnosed as ALL and must not receive more than 1 week of treatment other than VCR, glucocorticoids, and mediastinal irradiation [28, 43]. The criteria for risk stratification were similar to those of study XIII.

The pre-induction phase was 2 days of 6MP before the induction phase with LDMTX or HDMTX followed by induction therapy of 6 weeks, the same as XIIIB. After complete remission, the consolidation phase was started with HDMTX (5 g/M^2^) for high-risk patients and 2.5 g/M^2^ for low-risk patients. The continuation phase was the same as XIIIB with minor HDMTX dose modification: 2.5 g/M^2^ for low-risk patients and 5 g/M^2^ for high-risk patients. In the reinduction phase on days 1 and 8, the polyethylene glycol (PEG)-conjugated ASP and idarubicin were given and, on day 15, PEG-ASP only in addition to VCR every week and daily DEX for 120 weeks for girls and 146 weeks for boys. The CNS-directed included 16 doses of TIT for low-risk patients and 23 doses for high-risk patients initially infiltrated with CNS leukemia. There was no cranial irradiation for any patient either with CNS disease or not [27, 28, 43].

This study was exposed to early termination during remission induction due to toxicities and adverse event effects, so the outcome of study XI (*N* = 53 patients) had a 5-year OS estimate of 81.1 ± 5.3% and 10-year OS of 79.2 ± 16.1%. Also, 5-year EFS was 77.4 ± 5.7%, and 10-year EFS was 77.4 ± 16.5% [28]. In study XIV, only two isolated or combined hematological relapses with 10-year cumulative risk have occurred at 4.0 ± 2.8% [28]. One of the most important prognostic factors is early treatment response, and its evaluation depends on morphological examination of bone marrow or the level of MRD by flow cytometry must be less than < 0.0, the induction failure status when BM ≥ 5%, and MRD ≥ 1% which has been a significant prognostic factor [38].

## Total Therapy Study XV

The study aimed to boost the cure rate and enhance the quality of life by increasing the EFS, restricting anthracyclines and CYC doses, and omitting the cranial irradiation for all patients [37]. Participants (*N* = 501; age = 1–18 years) diagnosis of non-B-cell ALL was carried out by immunophenotyping, as determined by the reactivity pattern to a panel of monoclonal antibodies with flow cytometry as well as morphology and cytochemical staining. Patients previously treated with chemotherapy for 1 week or longer were excluded [37]. The patients are assigned to three risk groups: low, standard, and high risk [39]. Low-risk patients include cases with B-cell precursor and leucocyte coin less than 50 × 10^9^/L (age from 1 to 9.9 years) and more than or equal to 1.16 DNA index, or TEL-AML1 fusion, and patients with BM < 5% at 19 or 26 or MRD < 0.01% at the end of induction. Standard-risk cases were T cell or more than 50 × 10^9^/L (age ≥ 10 years) or B cell with testicular or CNS leukemia, hypodiploid (< 45 chromosomes), E2A-PBX1 fusion, or MLL rearrangement or patient with BM ≥ 5 at days 19 or 26 or 1 > MRD ≥ 0.01% at the end of induction. High-risk patients were those with t(9; 22) *BCR-ABL* fusion or patients with BM ≥ 5 (induction failure) or MRD ≥1% at the end of induction or MRD ≥ 0.1% at week 7 cases [39].

Treatment consisted of three main phases: remission induction, consolidation, and continuation. All patients received IT therapy on day 1 of the treatment. For phase I, they received PRD, VCR, DAN, L-ASP, CYC, CYT, and 6MP. Imatinib was administered from day 22 of induction if the patient was Ph-positive. Meanwhile, the IT therapy was initiated on days 1 and 19 with 2 additional doses for high-risk patients on days 8 and 26. The total numbers of IT treatments were 13 doses for (low-risk cases with CNS1 status), 18 (low-risk cases with CNS2 status, traumatic CSF with blast status, or WBCs > 100 × 10^9^/L), 16 (standard-risk cases with CNS1 status), 18 (standard-risk cases with CNS2 status), and 24 (standard-/high-risk cases with WBCs > 100 × 10^9^/L, presence of Ph chromosome, MLL rearrangement, hypodiploidy < 45, or CNS3 status). Moreover, CNS irradiation is removed except in (a) refractory CNS leukemia at diagnosis (patients with persistent blasts after 3 IT treatments): 2400 rad cranial irradiation with five TIT (with leucovorin rescue) at 1 year of remission; (b) patients with evidence of lymphoblasts in the CSF confirmed by immunological testing in continuation treatment on two separate occasions: systemic reinduction and four consecutive IT treatments, followed by CNS irradiation indicated as (i) patients with WBCs < 5/μL of CSF at first 18 months of initial remission: cranial irradiation (2400 rad in 16 fractions); (ii) for those with any number of leukemic lymphoblasts in CSF after 18 months of initial remission: cranial irradiation (1800 rad in 12 fractions); and (iii) for patients with WBCs > 5/μL of CSF occurring within the first 18 months of remission: craniospinal irradiation (2400 rad cranial irradiation in 16 fractions plus 1500 rad spinal irradiation in 10 fractions).

Concerning the consolidation phase, HDMTX and 6MP were administered daily in the first 56 days. The continuation phase included several schemes according to the risk category and reinductions I and II (Table 1) [41].

Five-year OS was 93.5 ± 1.9% and 5-year EFS 85.6 ± 2.9%. Also, 5-year cumulative CNS relapse was 2.7 ± 0.8% [39]. The relapse incidents were shallow, 15 patients had a hematological relapse, and 5 had CNS relapse and only one testicular relapse.

## Total Therapy Study XVI

Total therapy XVI assessed the benefits versus risks of a higher dose of PEG-ASP during the continuation phase, determined the EFS and OS relative to risk stratification, and enhanced the sequel of CNS relapse in high-risk children by intensifying TIT [41, 42]. Six-hundred patients were enrolled in the study with (1) confirmed precursor B cell and T cell, (2) patients ≤ 18 years, and (3) no previous treatment and if present is very restricted to 1 week or less systemic glucocorticoids, only single VCR dose, mediastinum radiation therapy, and a single dose of IT chemotherapy [41].

Patients were assigned to three risk groups: low-risk patients include cases with B-cell precursor and leucocyte coin less than 50 × 10^9^/L (age from 1 to 10 years) and more than or equal to 1.16 DNA index, or TEL-AML1 fusion with no testicular or CNS leukemia hypodiploid (< 45 chromosomes), E2A-PBX1 fusion, or MLL rearrangement or the translocation t (12; 21). High-risk patients were those with Ph chromosome or KMT2A rearrangement or early T-cell precursor, and standard risk is all others, including all T-cell ALL cases [41].

The treatment design was similar to that of XV with few modifications in the induction phase; IT therapy would be administered on days 1 and 15, dose-age dependent. Patients with a high risk of CNS relapse would receive 4 additional IT treatments on days 4, 8, 11, and 22. So, the total IT therapy will be determined according to risk status (13 doses for low-risk cases with CNS1 status), 17 doses for low-risk cases with CNS2 status, traumatic CSF with blast status, or WBC > 100 × 10^9^ /L), 16 doses (standard-risk cases with CNS1 status), 20 doses (standard risk cases with CNS2 status), and 27 doses (standard/high-risk cases with WBCs > 100 × 10^9^/L, presence of Philadelphia chromosome, MLL rearrangement, hypodiploidy < 45, or CNS3 status).

Moreover, the continuation therapy became more complex than XV (Table 1). Survival rate was quite high, 5-year OS of 94.1% (95% confidence interval (CI) = 91.7–96.5%) and 5-year EFS 88.2% (84.9–91.5%). Concerning the relapse incidents, eight patients had CNS relapse, and the 5-year cumulative risk of CNS relapse was 1.5% (95% *CI* = 0.5–2.5%) [41]. One patient had combined ocular and CNS, and one had combined hematologic and testicular. Finally, 26 patients had a hematological relapse [41].

## Total Therapy Study XVII

The study was designed in 2017 and still recruiting (estimated *N* = 1000; estimated completion = 2028). It was designed to improve children’s survival rate and quality of life diagnosed with ALL and lymphoblastic lymphoma (LLy) using novel precision medicine strategies depending on acquired or inherited genomic features and targeted therapy approaches [44]. The primary objective of the study includes the following:

i. Improving the EFS of provisional SR or HR patients with MRD at day 15 or day 22 (≥ 5%) or at the end of remission induction (≥ 1%) by using immunotherapeutic and molecular approaches including TKIs or chimeric antigen receptor T-cell/blinatumomab for patients with refractory B-ALL or B-LLy and bortezomib, a proteasome inhibitor for patients lacking a targetable lesion.
ii. Improving overall treatment in patients with T-ALL or T-LLy by targeting specific genomic mutations or by adding of proteasome inhibitor bortezomib for patients having inadequate early response and without targetable lesions and by using nelarabine for those with lymphoma or leukemia cells in CSF at diagnosis or their MRD at the end of induction (≥ 0.01%).
iii. Determining the incidence and severity of acute VCR-induced peripheral neuropathy after decreasing VCR dosage in high-risk patients with centrosomal protein 72 (*CEP72*) TT genotype or by decreasing the duration of treatment of VCR in those with the genotype of *CEP72* C/C or C/T.

Moreover, secondary objectives included measuring the OS and EFS of children with ALL and LLy and determining the tolerability of ruxolitinib treatment regimens in patients with Janus kinase/signal transducer and activator of transcription (JAK-STAT) mutations.

The study included patients with B- or T-cell ALL or LLy diagnosed by immunophenotyping and between 1 and 18 years. Patients should not receive prior therapy or limited therapy, including one dose of VCR, one dose of IT MTX, emergency radiotherapy, or systemic corticosteroids for 1 week or less. Patients diagnosed with LLy had less than 25% blasts in the bone marrow and peripheral blood by flow cytometry or morphology.

Concerning the design of XVII, low-risk patients with B-cell ALL or Lly will start the induction phase (6 weeks), oral PRD (40 mg/M^2^), and DAN (1–2 doses based on peripheral MRD at day 8 in hyperdiploid or patients with ETV6-RUNX1. At the same time, standard-risk patients are administering two doses of DAN. Patients with ABL1-class fusion will receive dasatinib, and those with down syndrome are given blinatumomab. Standard-risk patients will be given ruxolitinib for those with JAK-STAT mutation or ALL patients with MRD ≥ 5% at day 15 or day 22, and Lly, who will not achieve complete response at the end of induction, and for all patients with ETP and T/M MPAL. After completion of the induction phase, patients will enter the following therapy phases: early intensification (for standard-risk patients, 4 weeks), consolidation (8 weeks), and continuation (120 weeks)

High-risk patients will receive immunotherapy chimeric antigen receptor T cell, and those who do not respond to CAR T cell will receive re-intensification therapy. Blinatumomab will be given to those who cannot receive CAR T cell or patients with Down syndrome. Targeting agents, dasatinib, ruxolitinib, and bortezomib, are given the same as standard-risk patients but are stopped in immunotherapy and re-intensification therapy.

Patients with T-ALL or TLLy, standard risk, will receive the induction phase with PRD and DAB. Patients who completed the induction phase would receive early intensification (4 weeks), consolidation (8 weeks), and continuation (120 weeks). In contrast, high-risk patients will receive re-intensification after consolidation instead of the continuation phase. Dasatinib, ruxolitinib, and bortezomib are given as done for a patient with B-ALL/LLy standard risk but discontinued during re-intensification in T-ALL/LLy high risk.

Patients with *CEP72* T/T genotype will be randomized to receive VCR either (1.5 mg/M^2^) or (1 mg/M^2^) at the beginning of week 1 in the continuation phase. Moreover, patients with *CEP72* C/T or C/C genotype will be randomized to receive VCR (2 mg/M^2^) except during reinductions I and II (3 weekly doses of VCR 1.5 mg/M^2^ will be administrated) and pulse doses of DEX though the week of 49 or 101 of continuation phase.

## Discussion

In this review, we have reported chronologically the history of childhood ALL protocols development. According to contemporary childhood ALL studies [45, 46], childhood ALL has shown significant progress in survival in the last decade, with an OS rate exceeding 90% at 5 years and 94.3% OS for St. Jude Total Therapy XVI. This remarkable progress can be attributed to optimizing therapy according to the risk stratification criteria [47] and increasing chemotherapy intensity to the tolerance limit [48]. The recent clinical trials emphasize improving patients’ quality of life and avoiding long-term sequelae besides increasing cure rates [41].

Initially, the backbone of Total Therapy studies was designed based on the combinational therapy concept to reduce the chemotherapeutic resistance through early eradication of leukemic cells [46]. However, the balance between drugs’ safety and efficacy was crucial; for example, Total Therapy IV showed that reducing chemotherapy doses reduced the survival rate and shortened the duration of continuous complete remission [12]. In the context of safety, St. Jude used VM26 or VP16 plus CYT in the remission induction phase in Total Therapy XI, which caused a high incidence of therapy-related AML. Therefore, VP16 was removed in Total Therapy XV and was administered only during the intensification phase for high-risk patients [41]. Generally, the long duration of the continuation phase and intermittent intervals (4 weeks) of chemotherapy doses, besides variability in the strength of chemotherapy between different patients, decreases the therapy-related toxicity in low-risk patients.

The radiotherapy doses were increased gradually from Total Therapy I (500 rad) until Total Therapy V (2400 rad) to reach the maximum effect to eradicate the infiltrated leukemic cell in CNS, but it was insufficient. That was observed in the variability in the result of CNS relapse in Total III (50%) in comparison with Total V (9.3%) [7]. The Total VII proved that adding IT concurrent with radiotherapy together successfully eradicated the CNS leukemia more than radiotherapy alone; however, there was an obstacle, the radiotherapy-related toxicities considered as a challenge to balance the risk-benefit ratio of radiotherapy [6]. In 1991, during Total Therapy XII time, Neglia et al. reported that brain tumors were considered the most common secondary malignancy in survivors of childhood ALL [49]. At 15-year follow-up, Total Therapy X was significantly (*P* = 0.005) associated with higher incidences of secondary brain tumors (SBTs) than Total Therapies IX and XI (1.47%, 0.49%, and 0%, respectively). Despite the similarities in these protocols, SBTs were detected only in irradiated patients who presented with CNS leukemia at diagnosis, and higher doses of irradiation were associated with higher risk of secondary malignancy and high-grade tumors specifically. During follow-up, the median latency from the initial diagnosis of ALL to the diagnosis of SBTs was 12.6 years, and SBTs were inversely proportional to irradiation doses [21].

Recent studies confirmed that radiotherapy is a risk factor for second malignant neoplasms (SMN) induction [50]. Youlden et al. [51] analyzed data from the Australian Childhood Cancer Registry (1983–2013) and revealed that 388 (2%) of the children ≤ 14 years old developed SMN; its risk was fivefold higher among childhood cancer survivors compared to the general population [(standardized incidence ratio (SIR) = 5.13; 95% *CI* = 4.65–5.67)]. Abrahão et al. reported similar results in American children with ALL [50] using data from the American Surveillance, Epidemiology, and End Results 9 program (SEER 9) (1983–2014). They concluded that 578, (2.6%) of the children developed SMN (*SIR* = 5.51, 95% *CI* = 5.07–5.98). In comparison with other protocols, St. Jude Total Therapy XV showed a 5-year EFS of 86%, compared with 81% in the Dutch Childhood Oncology Group protocol (DCOG) ALL-9. Both studies have omitted the prophylactic cranial irradiation and used TIT (MTX, hydrocortisone, and CYT). St. Jude demonstrated an isolated CNS relapse of 2.7% and a combined CNS relapse of 1.2% compared with 2.6% and 2% for DCOG, respectively. Therefore, through Total Therapy XVI, St. Jude improved EFS and CNS control by intensifying IT therapy to reach its maximum benefits [41]. A recent review by Al-Mahayri et al. [52] highlighted that chemotherapeutic agents are independent risk factors SMNs induction. For example, anthracyclines target the two isoforms of topoisomerase II (Top2): Top2α and Top2β. Even though interfering with Top2α is the primary use for administering anthracyclines to suppress cell proliferation and replication, interfering with Top2β was associated with secondary tumors and cardiotoxicity. Also, VCR is the primary neurotoxic agent used in St. Jude protocols. It binds to the β-subunit of tubulin and inhibits microtubule formation, which are cytoskeletal proteins involved in several important cell functions such as cell shape, cell division, and chromosome segregation. Therefore, VCR causes acute peripheral neuropathy, and it is uncertain whether this effect continues to be experienced chronically [53].

The effect of HDMTX was observed in Total X. Therefore, the early intensification therapy followed by consolidation therapy of HDMTX Plus 6MP in Total XI provided remarkable enhancing in 5-year EFS from 53 to 71% [6]. According to Howard et al. [54], exposure to millimolar concentrations of MTX for minutes to hours may lead to acute renal, central nervous system, and liver toxicity. Chabner and Young [55] identified MTX threshold concentrations; exposure to 0.01 and 0.005 μM for over 24 h may result in the bone marrow and gastrointestinal epithelial toxicity, respectively. Hence, HDMTX may result in enhanced cytotoxicity due to abundant intracellular polyglutamation of the metabolite 7-OH-MTX. In 1980, when St. Jude was developing Total Therapy X, clinical trials estimated the incidence of renal dysfunction following HDMTX to be 1.8%, and the mortality among patients who developed renal dysfunction was 4.4% [56]. Therefore, in Total X, several clinical practices were adapted to minimize the toxicity of HDMTX. First, oral leucovorin with IV hydration (5% dextrose and 0.2% NaCl) and urinary alkalinization (NaHCO_3_) were given 2 h before each HDMTX infusion. Then, IV leucovorin was injected 12 and 18 h after MTX infection (Table 1).

Moreover, L-ASP formulation was believed to affect the treatment outcome, and it was replaced by PEG-ASP that has a longer half-life and is potentially less immunogenic. However, recent research shows that L-ASP and PEG-ASP demonstrate similar efficacy and safety [57]. Place et al. [58] reported that with a median follow-up of 6.0 years, the 5-year OS was 89% (95% *CI* = 85–93) for L-ASP and 90% (95% *CI* = 85–93) for PEG-ASP (*P* = 0.58). Still, IV PEG-ASP is recommended as the frontline ASP preparation in children with newly diagnosed ALL because it is associated with decreased anxiety compared with intramuscular L-ASP [58].

Risk stratification of patients was identified to develop risk-adapted treatment and refine therapy. In DCOG, pediatric patients with B-ALL are initially stratified as a standard risk with the following criteria: age ranged from 1 to < 10 years and WBCs < 50 × 10^9^ cells/L or stratified as high risk according to the following features: age > 10 years, WBCs > 50 × 10^9^ cells/L, testicular disease, CNS3, and chromosomal translocation t (9; 22) [59]. While in T-ALL, risk stratification according to DCOG is dependent on extramedullary diseases and status of MRD at day 29 of induction and consolidation for those who do not achieve remission at the end of induction [60]. The Dana-Farber Cancer Institute (DFCI) ALL Consortium stratifies patients at day 10 of induction based on results of next-generation sequencing panel, fluorescent in situ hybridization (FISH), and karyotyping [61]. In St. Jude Total Therapy XVII, *CEP72* polymorphism will be critical for patient stratification to personalized medicine for better outcomes. Although Gutierrez-Camino et al. [61] did not find an association between neurotoxicity during the induction phase and the CEP72 T/T genotype in Spanish children with ALL, other studies confirmed this relationship. Stock et al. [62] reported that *CEP72* T/T genotype frequency was higher in vincristine-induced neuropathy (31% vs. 10%, *P* = 0.022). Also, 75% of patients with *CEP72* T allele developed grades 2–4 neuropathy, compared to 44% of patients with CEP72 CC or CT genotype (*P* = 0.0221) [62]. In the Canadian Pharmacogenomics Network for Drug Safety (CPNDS) cohort, Wright et al. [63] reported that *CEP72* rs924607 T/T genotype was significantly associated with vincristine-induced peripheral neuropathies (odds ratio (OR) = 3.43; 90% *CI* = 1.15–10.3, *P* = 0.02). They also confirmed the results by a meta-analysis with other four studies (*OR* = 2.64; 95% *CI* = 1.62–4.31) [62, 64–66].

Currently, relapse appears only in up to 15% of the patients; however, death due to relapsed ALL remains one of the prominent causes of cancer mortality in children [67]. Analyzing St. Jude Total Therapy studies in the past 60 years shows that cytotoxic chemotherapy continues to be associated with both short- and long-term toxic effects. Therefore, risk stratification and devise targeted therapies can be improved by exploiting emerging molecular and immunologic insights to obtain optimal treatment plans.

## Conclusion

Throughout St. Jude’s journey for curing ALL, the goal was to minimize the chemotherapy’s adverse effect and achieve the highest cure rate and overall survival. Future research is targeting personalized therapy for better efficacy. The scientific and step-by-step problem-solving approach implemented in this journey might be helpful for other institutions in curing other serious diseases similar to ALL.

## Data Availability

Not applicable.

## Abbreviations

60Co: Cobalt-60
6MP: Mercaptopurine
ALL: Acute lymphoblastic leukemia
ASP: Asparaginase
CEP72: Centrosomal protein 72
CI: Confidence interval
CNS: Central nervous system
CPNDS: Canadian Pharmacogenomics Network for Drug Safety
CSF: Cerebrospinal fluid
CSI: Craniospinal irradiation
CYC: Cyclophosphamide
CYT: Cytarabine
DAN: Daunorubicin
DCOG: Dutch Childhood Oncology Group
DEX: Dexamethasone
DOX: Doxorubicin
EFS: Event-free survival
FAB: French-American-British
HDMX: High-dose methotrexate
IT: Intrathecal
IV: Intravenous
L-ASP: L-asparaginase
LLy: Lineage lymphoblastic lymphoma
MTX: Methotrexate
OR: Odds ratio
OS: Overall survival
PCP: *Pneumocystis carinii* pneumonia
PEG: Polyethylene glycol
PO: Oral
PRD: Prednisone
RBCs: Red blood cells
RTSC: Cranial irradiation/sequential chemotherapy
SBTs: Secondary brain tumors
SEER 9: Surveillance, Epidemiology, and End Results 9 program
SIR: Standardized incidence ratio
SMN: Second malignant neoplasms
t.i.d.: Three times a day
TIT: Triple intrathecal therapy
Top2: Topoisomerase II
VCR: Vincristine
VM26: Teniposide
VP16: Etoposide
WBCs: White blood cells
